# Fabrication and characterizations of simvastatin-containing mesoporous bioactive glass and molybdenum disulfide scaffold for bone tissue engineering

**DOI:** 10.1063/5.0172002

**Published:** 2023-12-04

**Authors:** Sesha Subramanian Murugan, Pandurang Appana Dalavi, Suprith Surya, Sukumaran Anil, Sebanti Gupta, Rohan Shetty, Jayachandran Venkatesan

**Affiliations:** 1 Biomaterials Research Laboratory, Yenepoya Research Centre, Yenepoya (Deemed to be University), Deralakatte, Mangalore, Karnataka 575018, India; 2 Advancement Surgical Skill Enhancement Division, Yenepoya (Deemed to be University), Deralakatte, Mangalore, Karnataka 575018, India; 3 Department of Dentistry, Oral Health Institute, Hamad Medical Corporation, College of Dental Medicine, Qatar University, Doha, Qatar; 4 Department of Surgical Oncology, Yenepoya Medical College Hospital, Mangalore, Karnataka, India

## Abstract

Due to the limitations of the current treatment approaches of allograft and autograft techniques, treating bone disorders is a significant challenge. To address these shortcomings, a novel biomaterial composite is required. This study presents the preparation and fabrication of a novel biomaterial composite scaffold that combines poly (D, L-lactide-co-glycolide) (PLGA), mesoporous bioactive glass (MBG), molybdenum disulfide (MoS_2_), and simvastatin (Sim) to address the limitations of current bone grafting techniques of autograft and allograft. The fabricated scaffold of PLGA–MBG–MoS_2_–Sim composites was developed using a low-cost hydraulic press and salt leaching method, and scanning electron microscopy (SEM) analysis confirmed the scaffolds have a pore size between 143 and 240 *μ*m. The protein adsorption for fabricated scaffolds was increased at 24 h. The water adsorption and retention studies showed significant results on the PLGA–MBG–MoS_2_–Sim composite scaffold. The biodegradation studies of the PLGA–MBG–MoS_2_–Sim composite scaffold have shown 54% after 28 days. *In vitro*, bioactivity evaluation utilizing simulated body fluid studies confirmed the development of bone mineral hydroxyapatite on the scaffolds, which was characterized using x-ray diffraction, Fourier transform infrared, and SEM analysis. Furthermore, the PLGA–MBG–MoS_2_–Sim composite scaffold is biocompatible with C3H10T1/2 cells and expresses more alkaline phosphatase and mineralization activity. Additionally, *in vivo* research showed that PLGA–MBG–MoS_2_–Sim stimulates a higher rate of bone regeneration. These findings highlight the fabricated PLGA–MBG–MoS_2_–Sim composite scaffold presents a promising solution for the limitations of current bone grafting techniques.

## I. INTRODUCTION

Bone is essential for movement, blood cell formation, and metabolic functions. Bone loss can affect physical activity and requires treatment.^1,2^ Autograft and allograft methods are used in orthopedics treatment, but each has advantages and disadvantages. Autograft offers histocompatibility and non-immunogenicity and needs extra surgery, while allograft offers sufficient resources but can transmit diseases. So synthetic materials are being explored to address these limitations. The researchers employed various techniques to fabricate a synthetic graft, including bioactive glasses, metals, synthetic biological materials, polymers, and ceramics.^2–4^ Synthetic graft materials are biodegradable, biocompatible, and nontoxic to human tissue.^3,5^ Mesoporous bioactive glass (MBG), a versatile biomaterial with an ordered mesoporous structure, was attractive for bone regeneration applications due to its biodegradability, clinical safety, and drug delivery capabilities.^6^ Electrospinning and surface doping procedures were used to enhance the surface characteristics and bioactivity of MBG-coated PLGA composite scaffolds, promoting human mesenchymal stem cell adhesion and proliferation.^7,8^

MBG and PLGA were coupled with vancomycin and fabricated a bone tissue-engineering scaffold that can be utilized to treat infected bone defects and chronic osteomyelitis. MBG facilitated mesenchymal stem cell attachment, proliferation, and osteogenic marker upregulation, leading to better cytocompatibility and osteoblastic differentiation. Vancomycin-loaded scaffolds effectively inhibited *Staphylococcus aureus* growth in infected bone, without impairing cytocompatibility.^9^ A recent study reveals an artificial cement of calcium sulfate powder, and strontium-containing mesoporous glass particles have 8 MPa compressive strength and promote bone repair mechanisms.^10^ Another study explores the potential of using lithium-containing mesoporous bioactive glasses (Li-MBG) to stimulate the activity of bone marrow stem cells (BMSCs) during the bone healing process under high-glucose conditions. Li-MBG was found to enhance integrin subunit alpha 3 (Itga3) and activate the β-catenin/Tcf7/Ccn4 signaling pathway, thereby reversing the adverse effects of high glucose and enhancing BMSCs proliferation and differentiation.^11^ Furthermore, poly-l-glutamic acid was mixed with MBG nanospheres and used in the drug delivery system for antibacterial activity. The results showed that the developed nanospheres had more antibacterial competence in gram-negative bacteria.^12^ Li *et al.* fabricated MBG–PLGA scaffolds containing fingolimod (FTY720) using supercritical carbon dioxide foaming. The results showed enhanced osteogenic differentiation and pro-angiogenic actions and improved vascularized bone regeneration on *in vivo* rat calvarial bone defect.^13^ MBG–PLGA was used for long-term controlled drug release of ibuprofen and egg white protein, demonstrating potential for bone healing applications.^14^ Liu *et al.* developed PLGA–MBG tri-hierarchical scaffolds using the combinations of PLGA and MBG through the salt leaching method. The macro–micro–mesopores in the scaffold significantly increased macrophage's M2 polarization and decreased M1 polarization, causing resonant fluctuation of pro- and anti-inflammatory genes and cytokines. *In vivo*, mice implantations showed increased neovascularization and ectopic bone development, and it also boosted the capacity for angiogenic growth and osteogenesis.^15^ Shi *et al.* created MBG–PLGA with chitosan composite (CS) scaffolds, demonstrating higher mechanical strength and mineralization capability than CS and pure PLGA-coated CS scaffolds. The MBG–PLGA/CS scaffolds also enhanced MC3T3 cell capacity for sustained drug administration, early differentiation, and cell proliferation.^16^ Song *et al.* utilized a supercritical carbon dioxide foaming technique to create MBGs and PLGA composite scaffold for tissue regeneration. The scaffold, with porosities ranging from 73% to 85% and sizes ranging from 120 to 320 *μ*m, demonstrated higher strength and Young's modulus than regular PLGA scaffolds. The scaffold's interconnecting macroporous structure promotes cell development by releasing bioactive ions to create a favorable environment for tissue engineering applications.^8^

MBG and molybdenum disulfide (MoS_2_) have recently drawn much attention because of their superior mesoporous structure, large surface area, and large pore volume. This accelerates biocompatibility, bio-inductivity, and conductivity behavior. Despite their high bioactivity, bioactive glasses and nano-MoS_2_ may not always exhibit the desired mechanical properties due to their brittle nature. As an alternative, a polymeric matrix is required for incorporation with an MBG and MoS_2_.^17^ The clinical application of MBG with MoS_2_–polymer composites has been limited, but these biomaterials offer advantages over traditional methods for delivering cells, drugs, and genes into the body. Simvastatin (Sim), an osteoinductive drug, promotes bone formation by stimulating osteoblastic activity and inhibiting osteoclastic activity.^18^ It improves bone mineral density and growth by stimulating the production of bone morphogenetic proteins (BMPs). The combination of PLGA–MBG–MoS_2_–Sim composites in a scaffold form may enhance mechanical properties, bioactivity, and drug release control. As a result, we aimed to fabricate the PLGA–MBG–MoS_2_–Sim composite scaffold for bone tissue regeneration and its applications in this work. For this purpose, we synthesized MBG and nanoMoS_2_ and fabricated the scaffolds using the salt leaching method. The MBG, MoS_2_, and developed scaffolds were characterized using x-ray diffraction (XRD), Fourier transform infrared (FT-IR), scanning electron microscopy (SEM), and transmission electron microscopic (TEM) analysis. The bone formation and regeneration capacity of the proposed scaffold were further examined in *in vitro* and *in vivo* models.

## II. RESULTS AND DISCUSSION

### A. Characterization of mesoporous bioactive glass

**FIG. 1.**
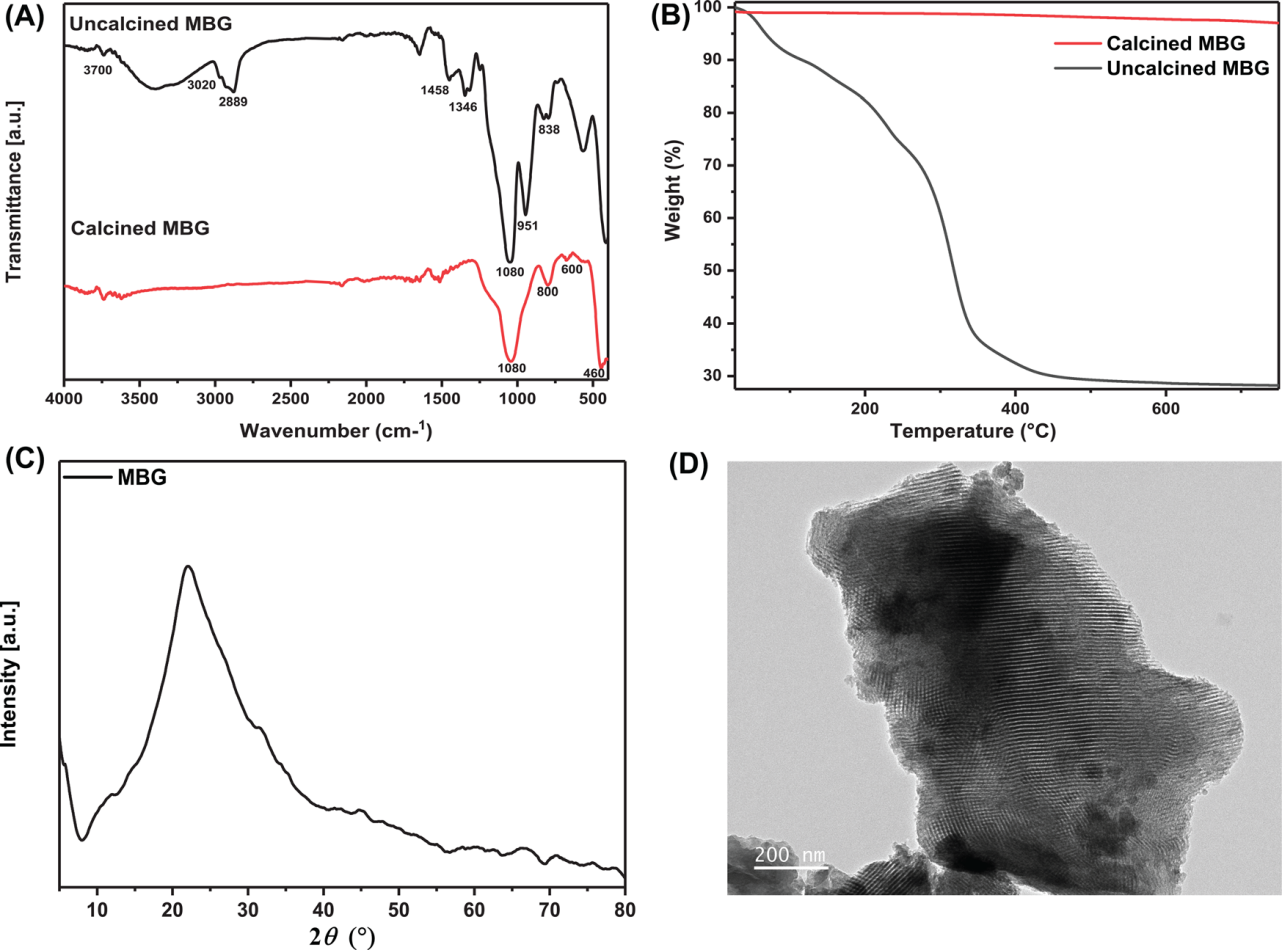
Structural characterization of MBG (a) FT-IR analysis of uncalcined and calcined MBG, (b) TGA analysis, (c) XRD spectra, and (d) TEM images with magnifications of 200 nm.

Figure 1 shows the structural characterization of MBG. The FTIR spectra for the synthesized MBG are demonstrated in Fig. 1(a). The uncalcined MBG has the characteristic O–H bending and C–H stretching bands of F-123 at 1342, 1346, and 2889 cm^−1^, respectively.^19^ Also, the peak ranges from 3700–3020 cm^−1^ correspond to O–H stretching or hydrogen bonds of adsorbed water.^20^ It is observed that peaks of F-123 and adsorbed moisture disappeared after the calcination of MBG.^21^ The calcined and uncalcined MBG spectra have the same Si–O–Si asymmetric stretching, bending, and rocking vibration at 1080, 800, and 460 cm^−1^. A band at 600 cm^−1^ corresponds to amorphous phosphate.^22^ Figure 1(b) shows the thermogravimetric (TG) analysis of MBG. On the TG curve for uncalcined MBG, three distinct zones of weight loss were seen. The uncalcined MBG shows the first area of weight loss (4.5%) in the temperature range between 30 and 100 °C. The moisture that has been physically absorbed may be eliminated up to 100 °C. The elimination of unreacted components, usually TEOS, may be the cause of the second region of weight loss (4.7%) for uncalcined MBG that is seen between 100 and 200 °C. Third weight loss (54.15%) was seen between 200 and 600 °C, showing the removal of Pluronic P123 used during the synthesis process. There were no discernible weight losses seen in the calcined MBG, suggesting that they were thermally stable.^23^ The XRD of MBG [Fig. 1(c)] shows 2θ peak range between 15° and 35° indicating the samples are amorphous in nature. The TEM morphology in Fig. 1(d) shows the mesoporous structure of MBG, and the pore volume calculated by the image J software confirms the results of 6.24 nm obtained by Brunauer–Emmett–Teller (BET) analysis, which is similar to previously reported literature.^24^ Also, the particle size of MBG was determined by dynamic light scattering (DLS) as 361 nm with the zeta potential of −13.48 mV, respectively. The elements of calcium, phosphorous, and silica were confirmed by x-ray photoelectron spectra (XPS). The atomic percentage of the MBG particles is O 1s—61.49%, Si 2p—29.56%; P 2p—1.03%; C 1s—5.03%; Ca 2p—2.89%. The results of high resolution XPS spectra for the MBG are shown in Figs. S1(a)–S1(d).

### B. Characterization of exfoliated MoS_2_

**FIG. 2.**
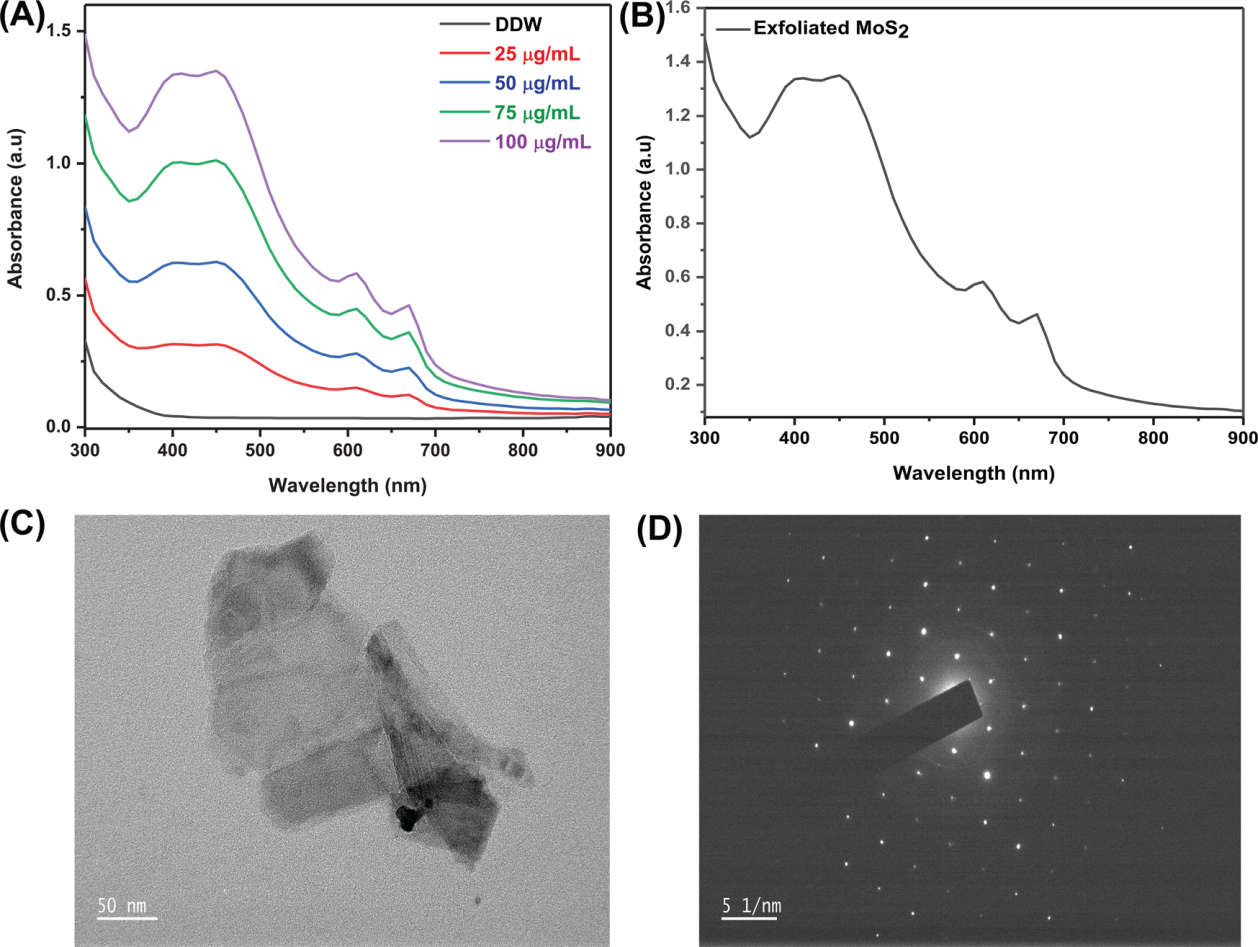
Morphology assessment of exfoliated MoS_2_ (a) UV spectra at different concentrations, (b) UV analysis of exfoliated MoS_2_, (c) TEM images with magnifications of 50 nm, and (d) SAED pattern of the MoS_2_.

The UV analysis in Figs. 2(a) and 2(b) confirms that MoS_2_ exfoliated from bulk powder. Figure 2(a) shows the exfoliated MoS_2_ absorption spectra at different concentrations; as the concentration rises, the absorption of the peaks also rises. Additionally, the findings support previously published research by indicating that exfoliated MoS_2_ nanosheet's absorbance had two peaks at 610 and 668 nm [Fig. 2(b)].^25^ The size and zeta potential of the exfoliated nanosheets were reported as 250 nm and −35 mV. Figures 2(c) and 2(d) show exfoliated nanosheet results from TEM and SAED (selected area electron diffraction). Flaked exfoliated nanosheets were found in the 230 nm dimensions range, and SAED patterns demonstrate the resulting exfoliated nanosheets were crystalline in nature.

### C. Characterization of PLGA–MBG–MoS_2_–Sim composites

**FIG. 3.**
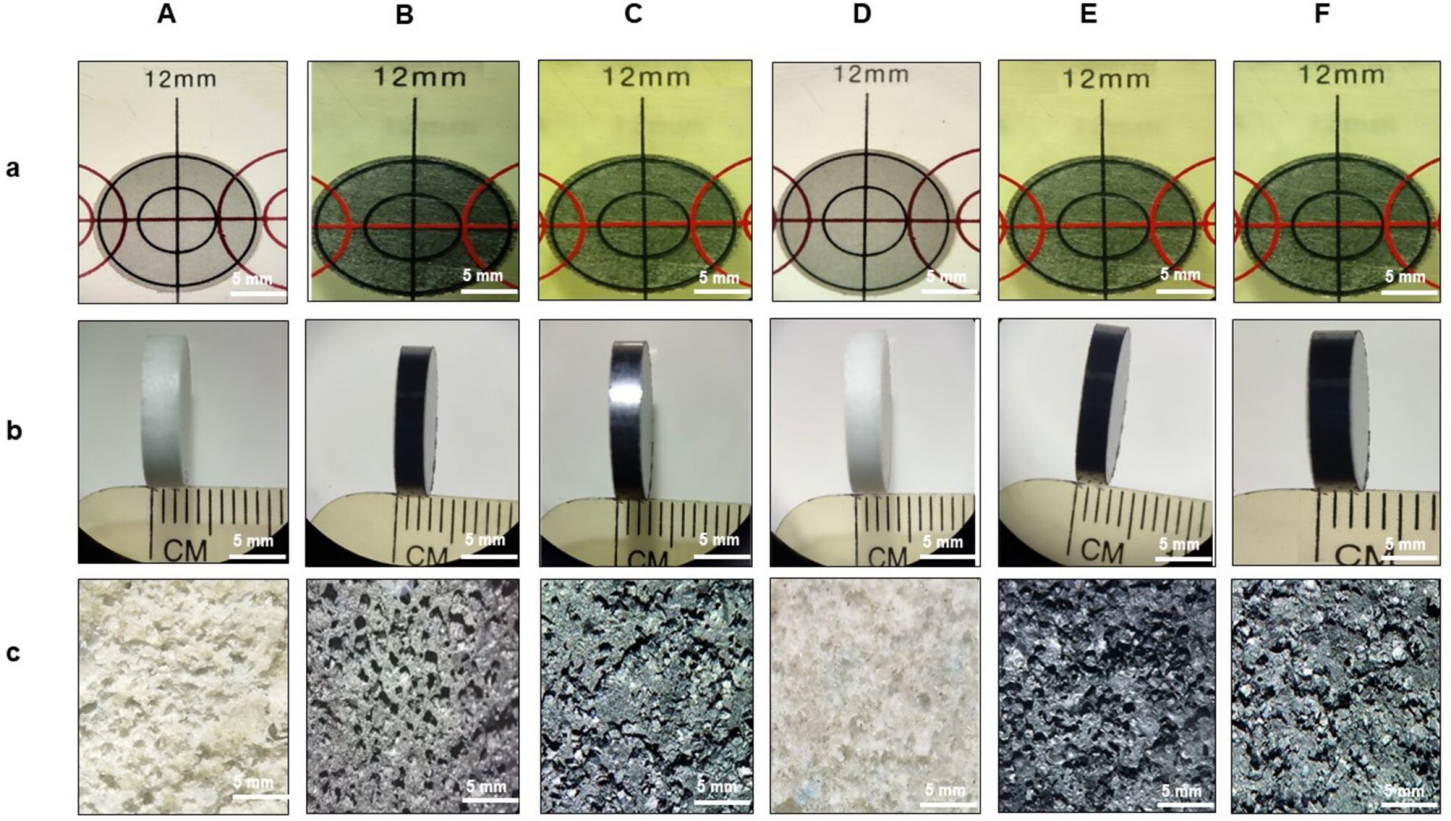
Microscopic analysis of PLGA-MBG/MoS_2_ composites. (a) PLGA–MBG, (b) PLGA–MoS_2_, (c) PLGA–MBG–MoS_2_, (d) PLGA–MBG–Sim, (e) PLGA–MoS_2_–Sim, and (f) PLGA–MBG–MoS_2_–Sim. (a) Diameter of composites, (b) thickness of composites, and (c) porosity nature of composites after salt leaching process.

The morphological observation of the developed composites is shown in Fig. 3 [(a) PLGA–MBG, (b) PLGA–MoS_2_, (c) PLGA–MBG–MoS_2_, (d) PLGA–MBG–Sim, (e) PLGA–MoS_2_–Sim, and (f) PLGA–MBG–MoS_2_–Sim]. The fabricated pellets were measured as 12.1 mm in diameter and thickness of 4 mm, as illustrated in Figs. 3(a) and 3(b). Also, the porosity nature of different composite pellets after salt leaching was well observed and is highlighted in Fig. 3(c).

**FIG. 4.**
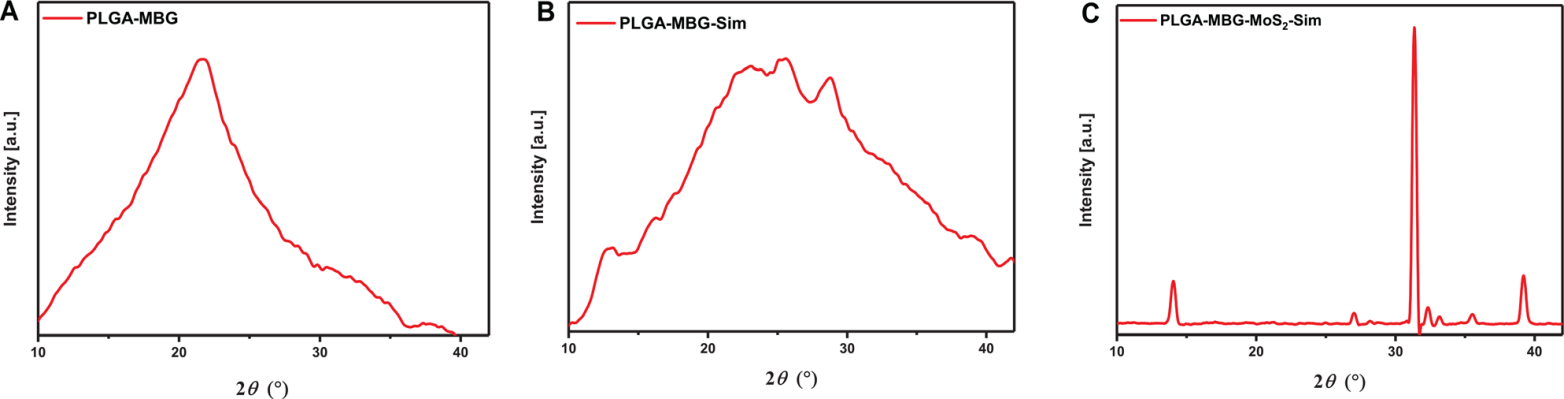
XRD analysis for the composite scaffold. (a) PLGA–MBG, (b) PLGA–MBG–Sim, and (c) PLGA–MBG–MoS_2_–Sim.

The functional groups present in the developed composites are demonstrated in Figs. S2 and S3. The structural characterization of PLGA–MBG–MoS_2_–Sim composites was examined using the XRD spectra in Fig. 4. In Fig. 4(a), the PLGA diffraction peak was located at 2θ = 10–26.47°.^26^ The relevant MBG diffraction peaks at 2θ = 15°–35° were identifiable in PLGA–MBG and PLGA–MBG–Sim composites.^27^ In PLGA–MBG–Sim composites [Fig. 4(b)], the intensity of the PLGA peaks decreased when the MBG was added to the composite matrix. Figure 4(c) demonstrates the existence of MoS_2_ diffraction peaks at 2θ region of 14.18°, 28.98°, 39.49°, 44.01°, 49.77°, and 60.08° in PLGA–MBG–MoS_2_–Sim, respectively. However, the characteristic peaks of the MBG were not visible in the XRD patterns of the PLGA–MBG–MoS_2_–Sim composite scaffolds. This is due to the overlap between the diffraction patterns of MBG and MoS_2_ in 2θ region. Additionally, the simvastatin peaks at 2θ regions of 8.22°, 10.81°, 14.82°,15.47°,16.39°, 17.12°, 17.57°, 18.69°, 19.28°, 21.93°, 22.52°, 25.80°, 28.24°, and 31.91° (Ref. 28) were overlapped with PLGA–MBG–Sim and PLGA–MBG–MoS_2_–Sim composite system. These XRD findings suggest that the PLGA–MBG has an amorphous character, whereas the addition of MoS_2_ and simvastatin provides an essential crystalline nature to the scaffold composite system for long-term osteoinductive and osteoconductive effects.^29^

The contact angle indicates the surface's wettability as well as the material's hydrophilic/hydrophobic character. A contact angle less than 90° suggests a hydrophilic surface, whereas a contact angle larger than 90° indicates a hydrophobic surface.^30^ Water contact angle for the different composite scaffold is shown in Table S1. The contact angles of the PLGA–MBG–MoS_2_–Sim composites were quite moderately hydrophilic, which offers a favorable environment for cells to adhere and proliferate.^31^

**FIG. 5.**
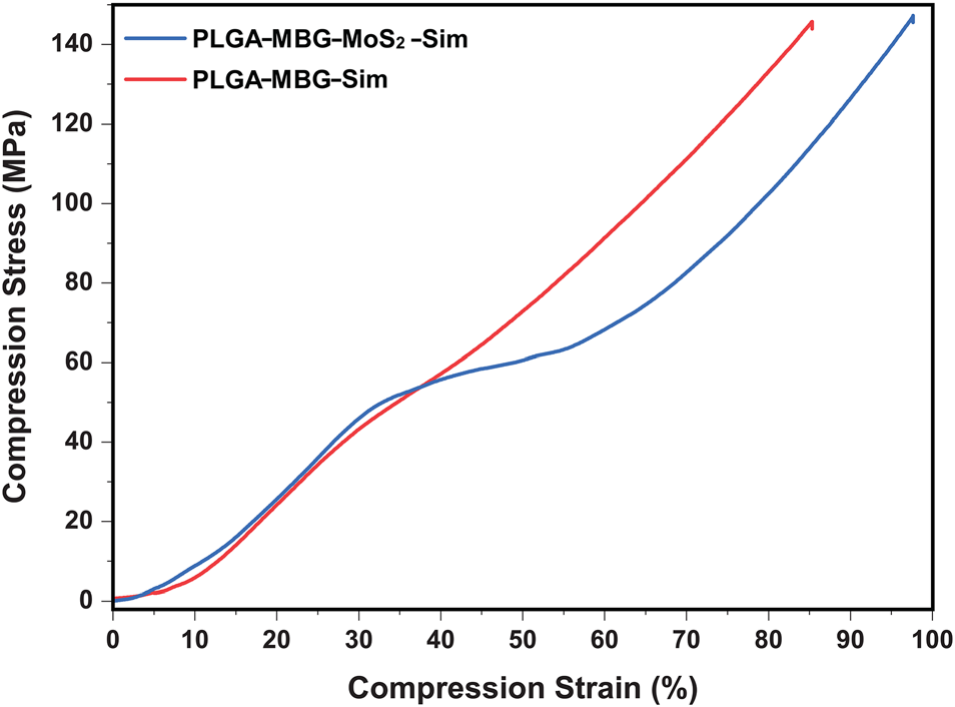
Mechanical strength of the PLGA–MBG–Sim and PLGA–MBG–MoS_2_–Sim composites scaffold.

Figure 5 depicts the mechanical strength of the PLGA–MBG–MoS_2_–Sim and PLGA–MBG–Sim. According to a prior study, pure MBG scaffolds had a mechanical strength of 60 kPa, which was increased to 250 kPa by adding 5% silk fibroin to MBG.^32^ Furthermore, Park and Kang *et al.* discovered that the compressive strength of PLGA was 48.1 ± 0.32 N.^33^ Appana Dalavi *et al.* estimated the mechanical strength of MoS_2_ microspheres to be 71.38 MPa.^25^ However, the mechanical strength of the composite scaffold of PLGA–MBG–MoS_2_–Sim and PLGA–MBG–Sim increased to 143 MPa, respectively. This might be due to PLGA linking the MBG and MoS_2_ and forming the uniform porosity network in the composite system after salt leaching. The compressive strength of cortical bone was 100–230 MPa,^34^ and hence, the PLGA–MBG–Sim and PLGA–MBG–MoS_2_–Sim fall within this range and thus imitate cortical bone.

**FIG. 6.**
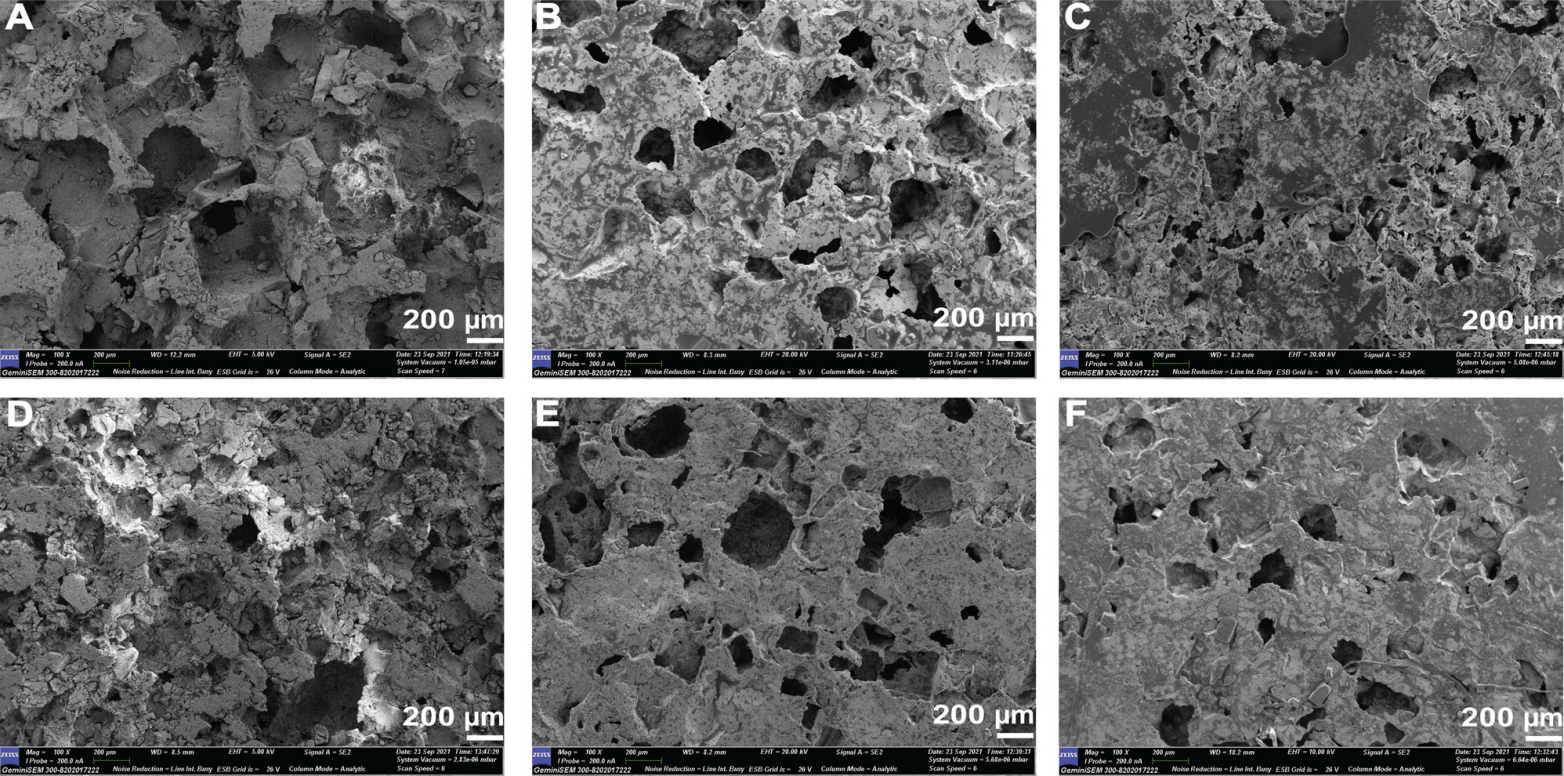
Morphology and pore size observation of composite scaffold. SEM image of 100× magnification (a) PLGA–MBG, (b) PLGA–MoS_2_, (c) PLGA–MBG–MoS_2_, (d) PLGA–MBG–Sim, (e) PLGA–MoS_2_–Sim, and (f) PLGA–MBG–MoS_2_–Sim.

**FIG. 7.**
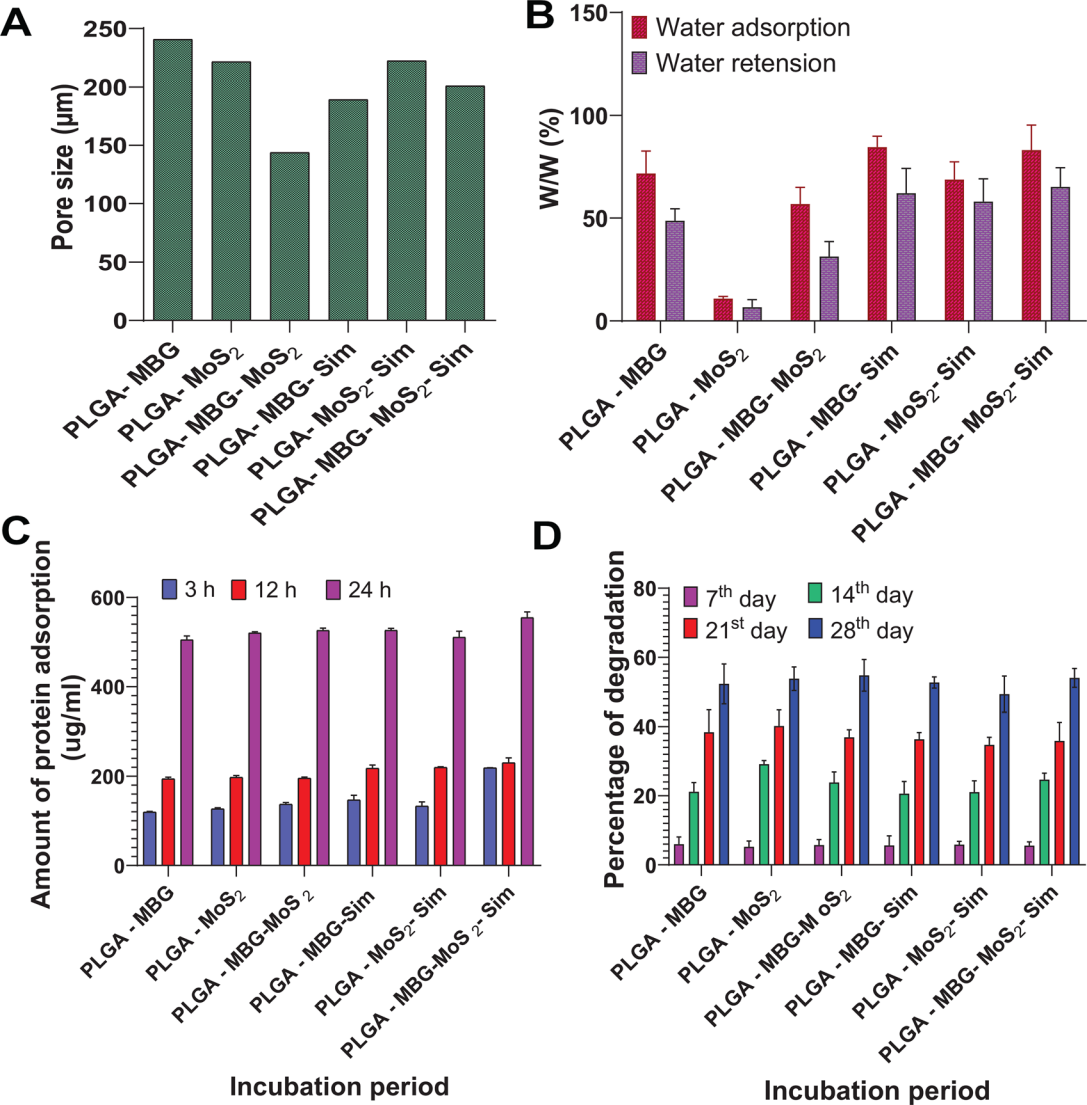
(a) Pore size, (b) water uptake and retention, (c) protein adsorption, and (d) bio-degradation behavior of composite scaffolds of PLGA–MBG, PLGA–MoS_2_, PLGA–MBG–MoS_2_, PLGA–MBG–Sim, PLGA–MoS_2_–Sim, and PLGA–MBG–MoS_2_–Sim.

Figure 6 shows the images of the developed composites. The addition of MoS_2_ to the composite system alters the morphology, texture, and pore size in the PLGA–MoS_2_, PLGA–MBG–MoS_2_, PLGA–MoS_2_–Sim, and PLGA–MBG–MoS_2_–Sim composite scaffolds. However, the pore size in the composite scaffolds of PLGA–MBG and PLGA–MBG–Sim was comparably high. The pore size of the scaffolds was found in the range between 143 and 240 *μ*m [Fig. 7(a)]. This agreed with the requirements of porous bone structure ranging from 20 to 400 *μ*m, necessary for the bone cells to adhere, multiply, and mature to new bone.^35^

Figure 7(b) depicts the water absorption and retention properties of developed composites. The results showed that there is a significant difference in water absorption behavior among the composite scaffolds, with water uptake being higher in the simvastatin-containing composites groups of PLGA–MBG–Sim, PLGA–MoS_2_–Sim, and PLGA–MBG–MoS_2_–Sim, compared to the control groups of PLGA–MBG, PLGA–MoS_2_, and PLGA–MBG–MoS_2_. It might be because of the incorporation of simvastatin, which increases the number of OH groups in the composite scaffold structure. Simvastatin-containing composites of PLGA–MBG–Sim, PLGA–MoS_2_–Sim, and PLGA–MBG–MoS_2_–Sim retained less water than the control groups PLGA–MBG, PLGA–MoS_2_, and PLGA–MBG–MoS_2_. This is because unbound water molecules were easily removed from the scaffold structure. Such adequate water uptake/retention ability of simvastatin-added scaffold composites may become helpful in the metabolic exchange of nutrients and blood flow for *in vitro* bone formation. The addition of MoS_2_ was shown to reduce both water adsorption and retention abilities in the control and simvastatin-treated groups.

Protein adsorption results are shown in Fig. 7(c). It was noticed that all composites showed increased protein adsorption with increasing incubation periods of 3, 12, and 24 h. Particularly, the PLGA–MBG–MoS_2_–Sim composite absorbs more protein than other composites might be due attributed to quite moderate hydrophilic nature. Furthermore, it is possible that the negatively charged surfaces of the precursor materials PLGA, MBG, and MoS_2_ might attract the positively charged amino acids in fetal bovine serum (FBS), which may enhance protein adsorption, triggering a cascade of cellular infiltration processes for bone cell growth.

Figure 7(d) depicts the biodegradation behavior of the developed composites. It was discovered that the degradation pattern in all composite systems follows the same trends and falls between 49% and 54% after 28 days. There was no significant difference in the degradation behavior of the composite system, which may be attributed to the same ratio of mixing the PLGA polymer with other components, and the polymer PLGA undergoes hydrolysis and degraded. This composite degradation rate may meet the demand for regeneration kinetics since bone mineral formation was observed in simulated body fluid (SBF) investigations after 28 days and bone formation was shown in x-ray analysis studies after 12 weeks in *in vivo* studies.

### D. *In vitro* bioactivity assessment of PLGA–MBG–MoS_2_–Sim composites

**FIG. 8.**
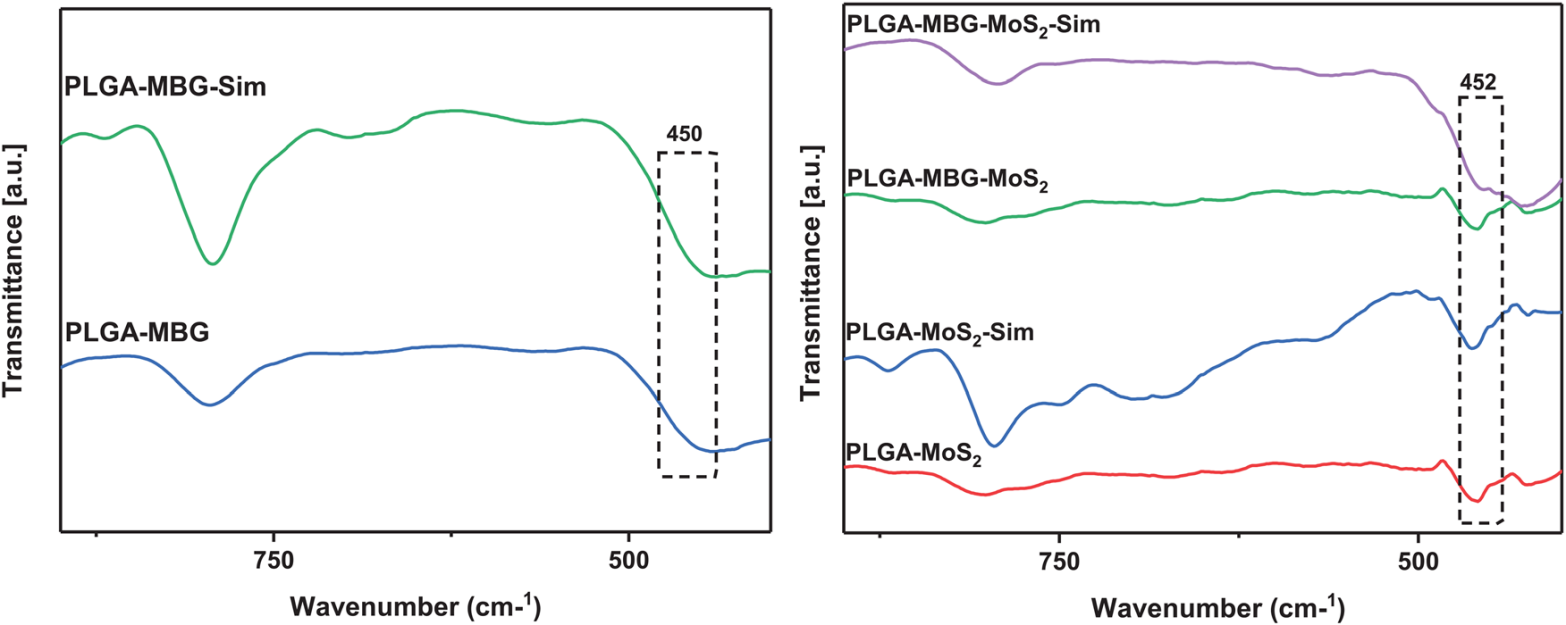
FT-IR spectral analysis of composite scaffolds after immersion in SBF for 28 days.

The development of hydroxyapatite is confirmed by FTIR analysis after soaking the PLGA–MBG–MoS_2_–Sim composites in SBF for 28 days. The spectra at an area 452 cm^−1^ were connected with the development of hydroxyapatite minerals, as shown in Fig. 8, which corresponds with recent reports.^36^ This demonstrates the potential of PLGA–MBG–MoS_2_–Sim composites to act as nucleating agents in hydroxyapatite formation.

**FIG. 9.**
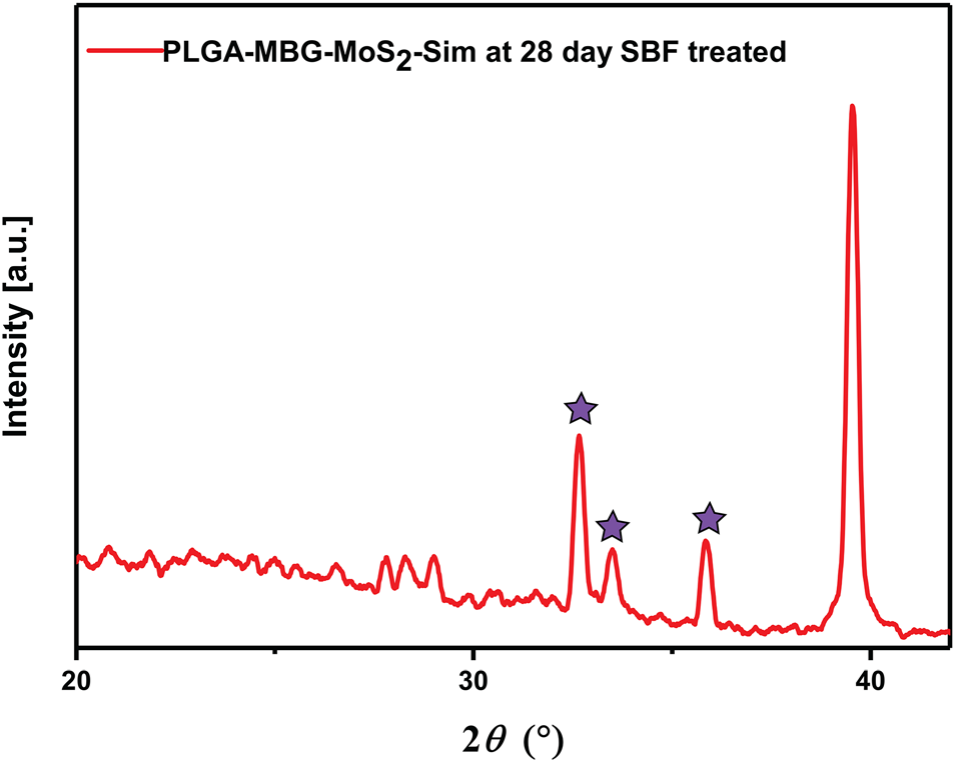
XRD assessment of PLGA–MBG–MoS_2_–Sim composite scaffolds after immersion in SBF for 28 days. The symbol star implies the 2Ø range for the formation of the hydroxyapatite minerals on the composite scaffold.

Additionally, the XRD analysis confirmed significant evidence for *in vitro* biomineralization following SBF treatment for 28 days. The equivalent peaks for hydroxy apatite were identified at 2θ regions of 32.7°, 33.52°, and 35.95° in PLGA–MBG–MoS_2_–Sim composites (Fig. 9).^37^ An earlier research has shown that MBG could develop bone minerals and establishes the development of hydroxyapatite peaks at 26.18°, 28.55°, 32.51°, 40.03°, 44.09°, 46.96°, 49.93°, and 53.68°.^23^

**FIG. 10.**
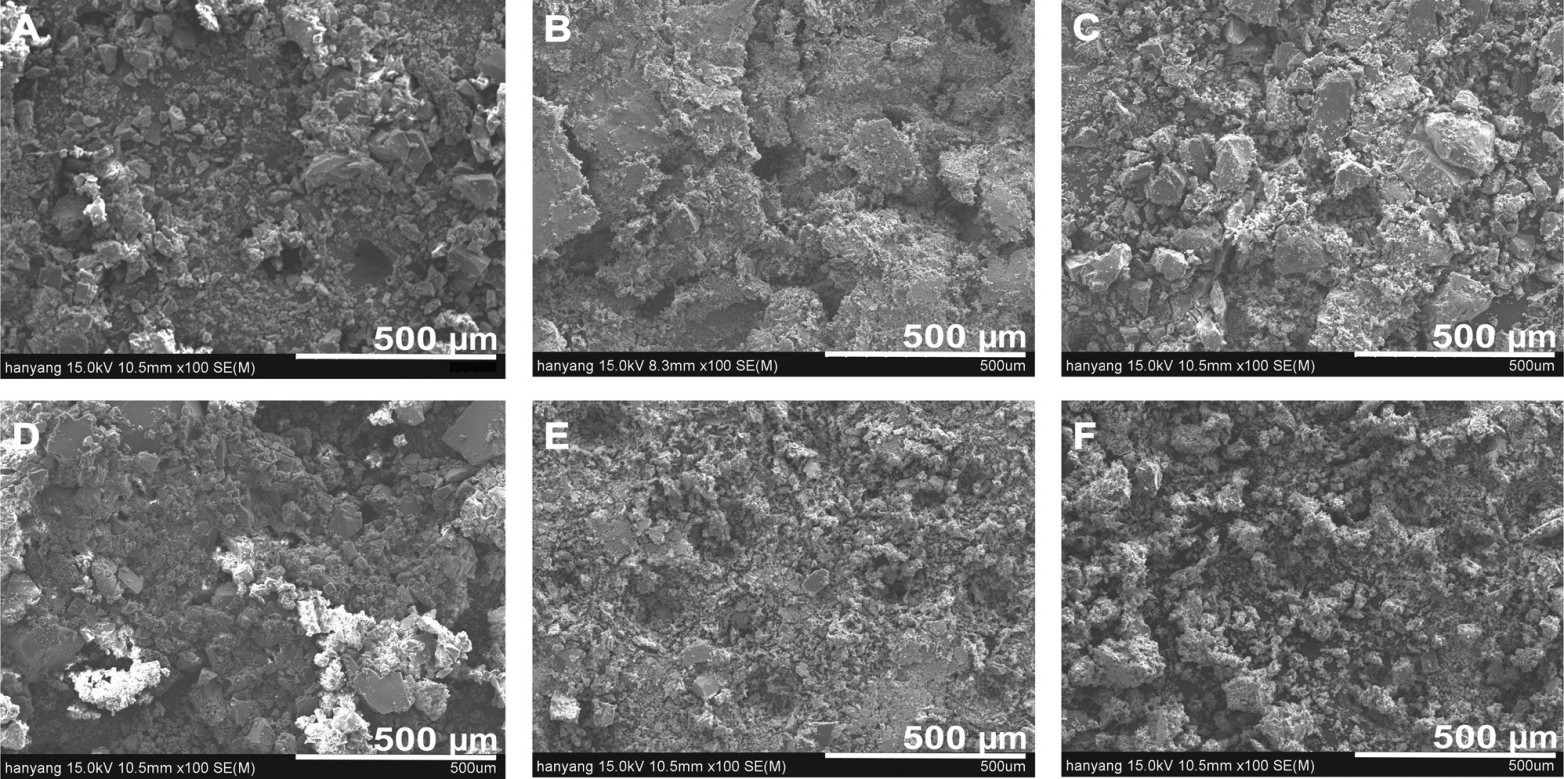
SEM image for observing hydroxyapatite formation on composite scaffolds after immersing in SBF for 28 days at 100× magnification. (a) PLGA–MBG, (b) PLGA–MoS_2_, (c) PLGA–MBG–MoS_2_, (d) PLGA–MBG–Sim, (e) PLGA–MoS_2_–Sim, and (f) PLGA–MBG–MoS_2_–Sim.

Figures 10(a)–10(f) show SEM micrographs of the PLGA–MBG–MoS_2_–Sim composites after 28 days of SBF soaking. Each sample's surface contains a spherical hydroxyapatite layer,^38^ which confirms the results of the XRD and FT-IR investigations. The scaffold structure collapsed after 28 days due to PLGA polymer breakdown, and morphology was examined using a powder sample. Furthermore, no hydroxyapatite development was seen in PLGA–MBG–MoS_2_–Sim composites before SBF immersion (Fig. 6), indicating mineral formation after SBF immersion. As a result, the developed PLGA–MBG–MoS_2_–Sim composites are considered to have a significant apatite forming potential.

### E. *In vitro* cytotoxicity, live/dead cell, alkaline phosphatase, and alizarin red staining

**FIG. 11.**
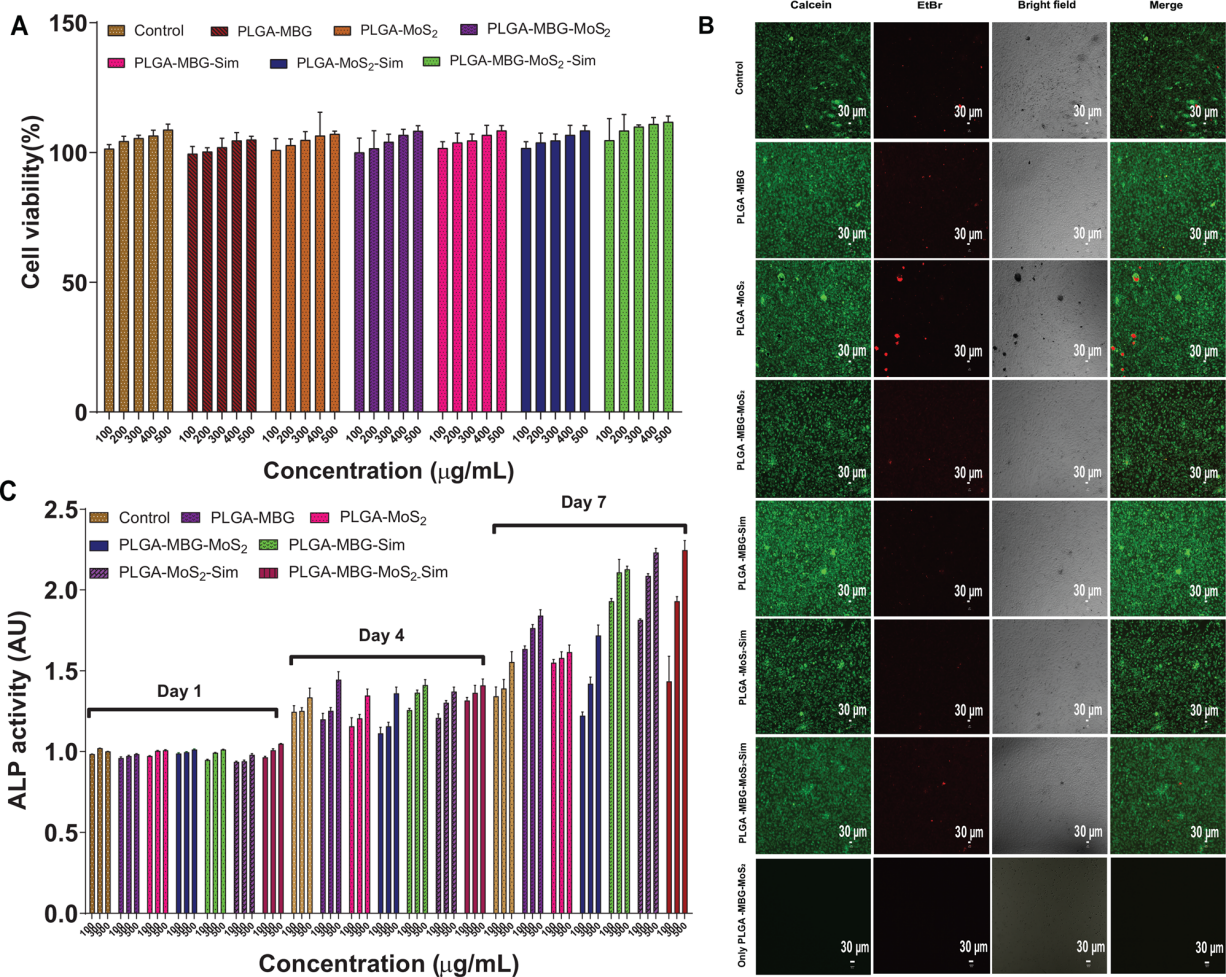
(a) The cell viability, where C3H10T1/2 cells were cultured for 48 h at 100, 200, 300, 400, and 500 *μ*g/ml of beta-tricalcium phosphate (control) and PLGA–MBG/MoS_2_ composites. (b) Live/dead cell staining of beta-tricalcium phosphate (control) and PLGA–MBG/MoS_2_ composites at 500 *μ*g/ml. (c) The alkaline phosphatase activity of beta-tricalcium phosphate (control) and PLGA–MBG/MoS_2_ composite at 100, 300, and 500 *μ*g/ml for 1, 4, and 7 days of incubation.

The WST-1 test was performed to assess the cytotoxicity of the PLGA–MBG–MoS_2_–Sim composites. The cytotoxic effects of C3H10T1/2 cells after 48 h of incubation on 100, 200, 300, 400, and 500 *μ*g/ml concentrations of control (beta-tricalcium phosphate) and PLGA–MBG–MoS_2_ composites are shown in Fig. 11(a). In every composite system, the results showed higher proliferation rate with respect to increasing concentration of samples as compared to the control group. This demonstrates that the PLGA–MBG–MoS_2_ composite system promotes cell proliferation. According to several research works, the MBG and MoS_2_ component helps in bone repair. Recent investigations on MBG have shown that rat bone marrow mesenchymal stem cells grow and differentiate into bone cells, reaching mineralization potential on day 14.^39^ Furthermore, Appana Dalavi *et al.* developed casein-coated MoS_2_ microspheres and investigated bone regeneration. At 250 *μ*g/ml concentration, exfoliated MoS_2_ had higher biocompatibility with MG-63, MC3T3-E1, and C2C12 cells and showed higher alkaline phosphatase (APL) activity and mineralization after 14 days.^25^ The live/cell staining shows the proportion of the live and dead cells within the treated composite samples. Figure 11(b) illustrates the confocal laser microscopic images of C3H10T1/2 cells stained with calcein EtBr solution being treated with 500 *μ*g/ml PLGA–MBG–MoS_2_–Sim composites. The live cells exhibit high-intensity green fluorescence in every composites, while dead cells exhibit significantly less red fluorescence. This result showed the biocompatibility behavior of PLGA–MBG–MoS_2_–Sim composites, as well as their influence on the viability of C3H10T1/2 cells. Furthermore, no noticeable fluorescence was observed on “Only PLGA–MBG–MoS_2_–Sim” composites at 500 *μ*g/ml “without cells,” confirming that biomaterials had no interaction (non-specific markage) with calcein and EtBr staining.

The ability of PLGA–MBG–MoS_2_–Sim composites to develop osteoblast cells from C3H10T1/2 cells was determined using alkaline phosphatase activity. ALP regulates inorganic phosphate movement during calcium formation and promotes cell division or differentiation. In the present study, the ALP activity was measured at concentrations of 100, 300, and 500 *μ*g/ml of the control (beta-tricalcium phosphate) and PLGA–MBG–MoS_2_–Sim composites. The results are shown in Fig. 11(c), and it revealed that ALP activity was significantly improved at every time point as well to increased concentration of control and PLGA–MBG–MoS_2_–Sim composites. On the seventh day, the PLGA–MBG–MoS_2_–Sim composite had higher ALP activity than the other composite systems and control. In addition, the activity was increased to 500 *μ*g/ml, and this concentration has been used for further experiments. According to several research studies, MBG^40^ and MoS_2_^41^ have enhanced ALP activity and bone repair and regeneration.

**FIG. 12.**
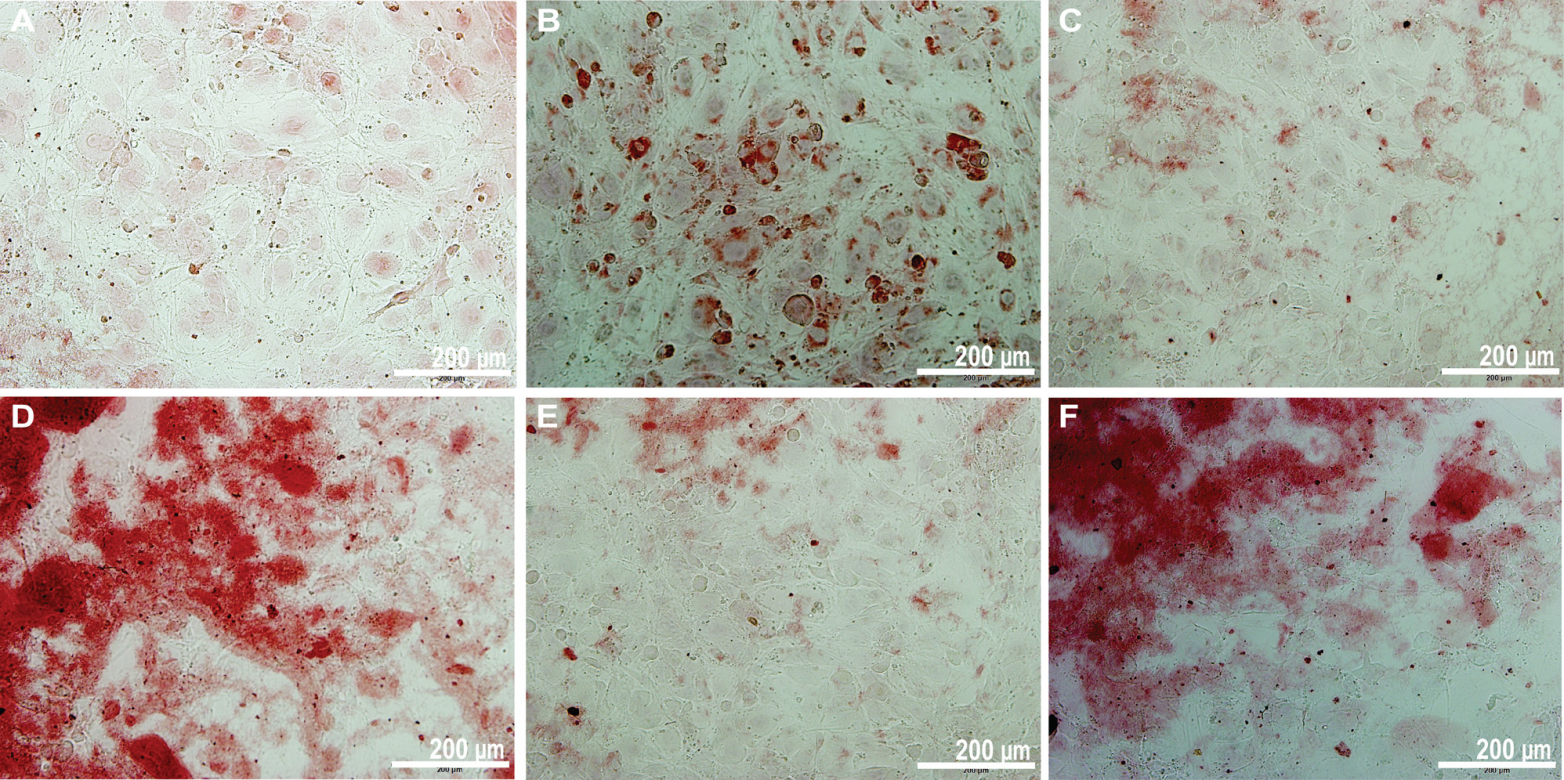
Analysis of mineralization potential of (a) blank, (b) control (beta-tricalcium phosphate), (c) PLGA–MBG, (d) PLGA–MBG–MoS_2_, (e) PLGA–MBG–Sim, and (f) PLGA–MBG–MoS_2_–Sim composites in which C3H10T1/2 cells were cultured for 7 days at 500 *μ*g/ml. Scale bar = 200 *μ*m. Magnification = 20×.

The primary approach for investigating osteogenesis is to figure out the biological process that directs the formation of bone minerals. The mineralization findings are crucial for understanding how biomineralization develops during osteogenesis in the new material. With C3H10T1/2 cells, we examined the effects of calcium mineral formation on blank [osteogenic differentiation media (ODM) + cells], ODM medium with control (beta-tricalcium phosphate), and PLGA–MBG–MoS_2_–Sim composites at 500 *μ*g/ml. The alizarin red s staining findings shown in Fig. 12. have deeper red calcium mineral staining over more extended incubation periods. Furthermore, the red staining on the PLGA–MBG–MoS_2_–Sim composites was deeper than blank, control, and other composite groups. As a result, the enhanced mineralization potential on PLGA–MBG–MoS_2_–Sim was found, and this composite has been used for further studies. Likewise, recent studies have reported that 3D printed PLGA/MBG has bone mineral formation over different time points and 28 days.^42^

### F. Regeneration of bone fracture: *In vivo* studies

**FIG. 13.**
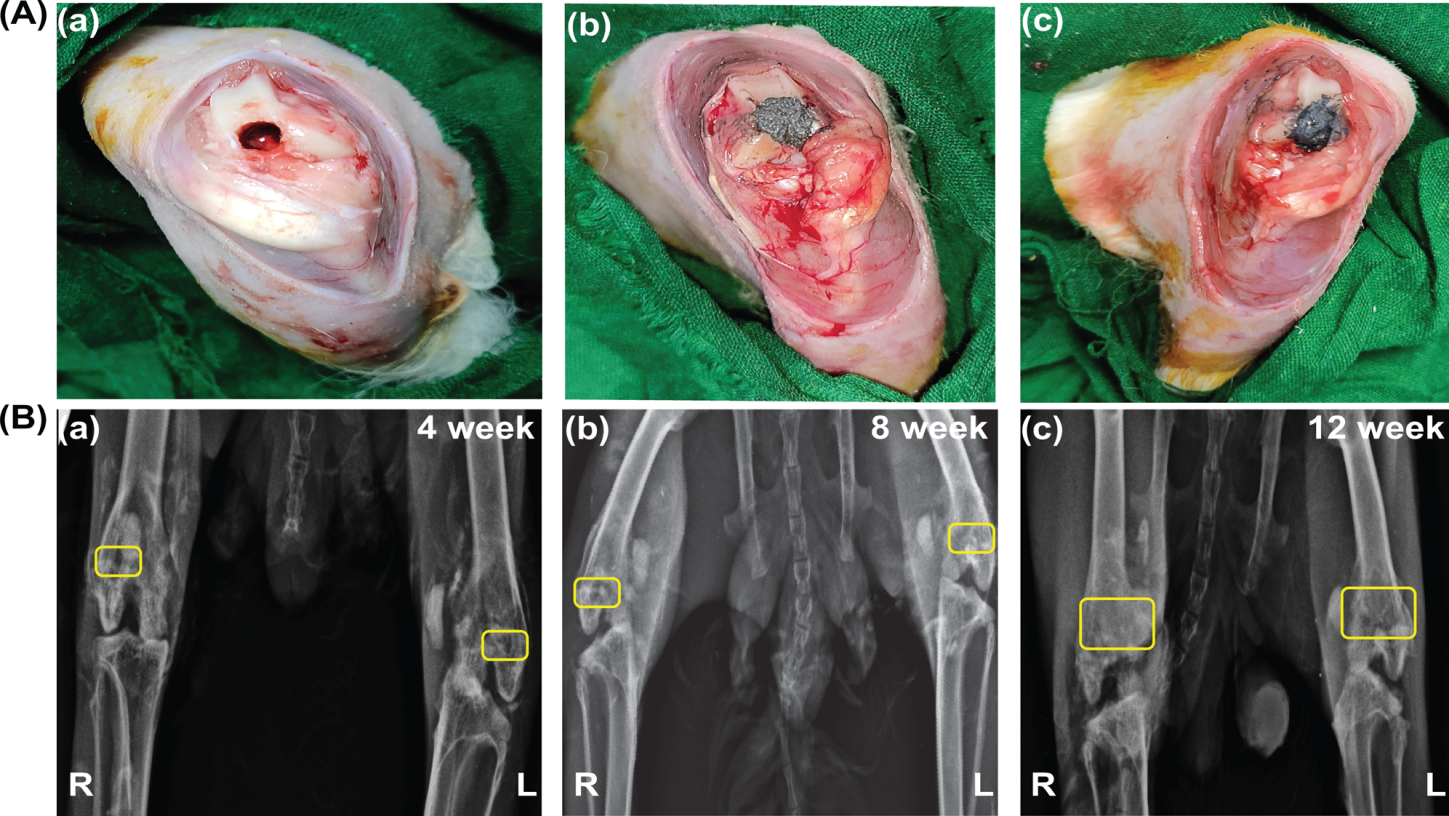
Images (A) femur condyle region of (a) Φ 5 × 5 mm^2^ defect; (b) implanted with control material (PLGA–MBG–MoS_2_); and (c) implanted with treated material (PLGA–MBG–MoS_2_–Sim). (B) X-ray analysis from right and left femur condyle region of rabbits. The yellow spots indicated the location of the defect and implanted material on the condyle femur defect. R depicts the right hind limbs, where the PLGA–MBG–MoS_2_–Sim composites were implanted. The PLGA–MBG–MoS_2_ was implanted in the left hind limb, as the letter L shows. The regeneration abilities were noted at various intervals throughout (a) fourth week, (b) eighth week, and (c) twelfth week.

A femur condyle region with critical size defect and implanted materials of PLGA–MBG–MoS_2_ and PLGA–MBG–MoS_2_–Sim are shown in Figs. 13(a-a), 13(a-b), and 13(a-c). After implanting the scaffolds in the rabbit femur condyle defect for 4, 8, and 12 weeks, x-ray examinations were performed at three different intervals to assess the osteointegration capacities of PLGA–MBG–MoS_2_ and PLGA–MBG–MoS_2_–Sim. The yellow mark in Fig. 13(b) displays pictures of an x-ray from a rabbit condyle femur defect implanted with PLGA–MBG–MoS_2_ on the left hind limbs and PLGA–MBG–MoS_2_–Sim on the right hind limbs. The x-ray study findings reveal the regeneration of the bone fracture around the implanted materials commencing at 4, 8, and 12 weeks. The wound size was still visible on the left and right hind limbs at the end of the fourth week [Fig. 13(b-a)], despite treatment with PLGA–MBG–MoS_2_ and PLGA–MBG–MoS_2_–Sim. In the eighth week [Fig. 13(b-b)], the defect size decreased on the left hind leg, while the wound get started closing on the right. Also, during the twelfth week [Fig. 13(b-c)], the defect size was closed entirely in the right hind limb location where the PLGA–MBG–MoS_2_–Sim materials were implanted, while it was significantly smaller in the left hind limb has PLGA–MBG–MoS_2_. This demonstrates PLGA–MBG–MoS_2_–Sim has accelerated more bone regeneration, bone tissue integrity, and wound healing than PLGA–MBG–MoS_2_.

## III. CONCLUSION

In the current study, we have developed PLGA–MBG–MoS_2_–Sim composites scaffold for bone tissue engineering. We have prepared MBG and MoS_2_ separately for the scaffold fabrication. First, the solvent evaporation method provided the basis for forming the MBG, and FTIR shows the presence of functional groups in MBG. Thermogravimetric analysis (TGA) confirmed the thermal stability of MBG. The 2θ range of 15°–25° of XRD ensures the formation of the amorphous nature of MBG. The DLS measurements indicate the synthesized MBG has 312 nm in size. BET and TEM analyses confirm the formation of the 6.24 nm mesoporous pore size. The elemental and x-ray photoelectron spectroscopy analysis confirmed the existence of calcium, phosphorus, and silica in MBG. The presence of calcium and phosphorous in MBG regulates the bioactivity behavior for osteogenesis. Furthermore, the liquid exfoliation method was used for the synthesis of nano-MoS_2_. The exfoliation of nano-MoS_2_ was validated by UV analysis, which indicated an absorption peak at 610 and 668 nm. Flaked exfoliated nanosheets with a diameter of 136 nm have been identified by TEM analysis. We used a simple hydraulic press approach to create a 12.1 × 4 mm^2^ PLGA–MBG–MoS_2_–Sim composite scaffold in our study. In comparison with other fabrication techniques, it was significantly less cost-effective and more rapid. However, compared to other composite systems, the water contact angle measurement for PLGA–MBG–MoS_2_–Sim revealed the moderate hydrophilic nature, with a value of 43.7°, which required for osteoblast cells to proliferate and differentiate. The mechanical strength of PLGA–MBG–MoS_2_–Sim was 143 MPa, which meets the demand for cortical bone strength. The XRD, FT-IR, and SEM results of the PLGA–MBG–MoS_2_–Sim before and after SBF immersion confirmed the generation of hydroxyapatite as well as the mineralization potential for the osteogenesis process. PLGA–MBG–MoS_2_–Sim composites also showed considerable protein adsorption and long-term biodegradation. In addition, the PLGA–MBG–MoS_2_–Sim composites demonstrated improved ALP activity, increased mineralization ability, and superior bio-compatibility in C3H10T1/2 cells. Therefore, the bone fracture model on the rabbit's femur condyle region shows that the PLGA–MBG–MoS_2_–Sim composite implant material stimulates bone regeneration through the healing process at the twelfth week, making it essential to use PLGA–MBG–MoS_2_–Sim composites effectively to connect the broken bones. These results imply that PLGA–MBG–MoS_2_–Sim composites may be suitable as a biomaterial for bone grafting.

## IV. METHODS

### A. Materials

Poly (D, L-lactide-co-glycolide) (Resomer^®^ RG 502 H and average Mw ∼7000–17 000) was given by Evonik (P) Ltd. Mumbai, India. Molybdenum (IV) sulfide (MoS_2_), Pluronic F-127, triethyl phosphate (C_6_H_15_O_4_P), tetra ethyl ortho silicate (TEOS) (SiC_8_H_20_O_4_), di-potassium hydrogen phosphate tri hydrate (K_2_HPO_4_·3H_2_O), sodium sulfate anhydrous (Na_2_SO_4_), potassium chloride (KCl), sodium chloride (NaCl), calcium chloride (CaCl_2_), potassium dihydrogen phosphate (KH_2_PO_4_), and hydrochloric acid (HCl) were purchased from Sigma-Aldrich (St. Louis, MO, USA). Ethanol was procured from analytical CSS reagent Changshu, China. Calcium nitrate tetrahydrate [Ca (NO_3_)_2_·4H_2_O] was obtained from Nice Chemicals (P) Ltd. Cochin, India. Sodium bicarbonate (NaHCO_3_) and magnesium chloride hexahydrate (MgCl_2_·6H_2_O) were provided by HiMedia, Mumbai, India. The drug simvastatin, β-glycerophosphate disodium salt hydrate, β-tri-calcium phosphate, dexamethasone, L-ascorbic acid, triton X-100, alizarin red s, fetal bovine serum (FBS), and penicillin–streptomycin were obtained from Sigma-Aldrich (St. Louis, MO, USA). WST-1 assay was obtained from Roche Diagnostics (Mannheim, Germany). Calcein AM and ethidium homodimer were supplied from Invitrogen (Carlsbad, CA, USA). High-glucose Dulbecco's modified eagle's medium (DMEM) and trypsin–disodium ethylene diaminetetraacetic acid (EDTA) solution were procured from Welgene (Daegu, South Korea).

### B. Synthesis of mesoporous bioactive glass

The MBG was prepared by the evaporation induced self-assembly (EISA) method.^43^ 16 g of Pluronic F127 (surfactant) was added to 240 ml of ethanol and agitated for 5 min at room temperature. After, 4 ml of 0.5 M hydrochloric acid was added to the above solution and stirred until the surfactant was dissolved and the clear solution was seen. Following, 29.60 g (31.70 ml) of tetra ethyl ortho silicate (TEOS), 2.72 g (2.5 ml) of triethyl phosphate, and 3.92 g of calcium nitrate tetrahydrate [Ca(NO_3_)_2_·4H_2_O] were added into the solution under continuous stirring of 3 h interval. After adding components, the solution was allowed to stir at room temperature for 24 h. Then, the resulting clear solution was transferred to the petri plates, allowing for the EISA process at 20 °C for 7 days. After completion of the EISA process, the obtained homogenous membranes were calcined in an air atmosphere at 700 °C.

### C. Synthesis of exfoliated molybdenum (IV) sulfide

The method to produce exfoliated MoS_2_ from bulk sources was followed as per protocol.^44^ In a particular experiment, 2 mg/ml of gum Arabic (GA) was dissolved in 40 ml of distilled water. Then, 5 mg/ml of bulk MoS_2_ was mixed with 40 ml of GA solution and sonicated for 1 h in ice cold (∼4 °C) conditions using a Qsonica probe sonicator. Following sonication, the solution was centrifuged at 5000 rpm for 30 min. The supernatant solution was collected and centrifuged for 30 min at 10 000 rpm. The exfoliated MoS_2_ was settled at the bottom as a residue and was collected by removing the supernatant. The collected residue was mixed with 10 ml of distilled water to disperse the exfoliated MoS_2_. After, the exfoliated MoS_2_ solution was freeze-dried overnight and used for further experiments.

### D. Fabrication of PLGA–MBG–MoS_2_–Sim composite scaffolds

0.4 g PLGA was added with 1.3 g NaCl and crushed into fine powder using a mortar and pestle. The fine powder was blended with 0.3 g MBG and 0.3 g MoS_2_ and crushed again until it resembled talcum powder appearance. Three types of composite mixture were prepared using PLGA–MBG, PLGA–MoS_2_, and PLGA–MBG–MoS_2_. To these composite mixtures, 10 mg simvastatin drugs were added separately and formulated into the different composite systems: PLGA–MBG–Sim, PLGA–MoS_2_–Sim, and PLGA–MBG–MoS_2_–Sim. The following steps were followed for the fabrication of different scaffolds. 1 g of the composite mixture was added to the pelleting die mold designed by PCI analytics, Thane, India. The load pressure was set to 2 MPa for 2 min, and the pellet scaffold was developed. This scaffold was heat incubated at 100 °C for 15 min.^45^ Following, the developed scaffold was taken in a 12 kDa dialysis membrane with distilled water, and the dialysis process was performed for 48 h. Following dialysis, the available NaCl in the scaffold was leached out, and porous nature was developed. Finally, the porous developed scaffold was heat incubated for 4 h and cooled under ambient conditions. The porous developed scaffolds were stored in a desiccator for further characterization.

### E. Chemical characterizations

The mentioned PLGA–MBG/MoS_2_ composites in the study include PLGA–MBG, PLGA–MoS_2_, PLGA–MBG–MoS_2_, PLGA–MBG–Sim, PLGA–MoS_2_–Sim, and PLGA–MBG–MoS_2_–Sim.

#### 1. Microscopic analysis

The structure, shape fidelity, and macro-porosity of the fabricated PLGA–MBG–MoS_2_ composite scaffold were observed using a stereomicroscope (ZEISS Stemi DV4, Germany). The images were captured using a mobile quad-camera at 108 megapixels.

#### 2. Fourier transform infrared spectroscopy analysis

The functional group related to uncalcined and calcined MBG, before and after immersion of SBF with PLGA–MBG–MoS_2_–Sim composites, were investigated in the spectral range of 4000–400 cm^−1^ by using Fourier transform infrared (FTIR) spectroscopy (Shimadzu, Kyoto, Japan). Attenuated total reflectance mode and 32 scans per acquisition were used for the spectral analysis.

#### 3. Thermogravimetric analysis

The thermal degradation characteristics of uncalcined and calcined MBG were investigated using a thermogravimetric (TG) analyzer (Make: TA Instruments, New York & Model: Q600 SDT). The samples were analyzed in a nitrogen (N_2_) environment at a temperature ranging from 20 to 800 °C at a heating rate of 10 °C/min.

#### 4. X-ray diffraction analysis

The Bruker system with Cu x-ray radiation was used to study the x-ray diffraction (XRD) spectra of plain and SBF-treated MBG, PLGA–MBG–MoS_2_–Sim composites.

#### 5. Dynamic light scattering analysis

The particle size of the MBG and exfoliated MoS_2_ were determined by dynamic light scattering (DLS) using a particle analyzer (Malvern Panalytical, Malvern, United Kingdom) at the temperature of 25 °C.

#### 6. Brunauer–Emmett–Teller analysis

The pore size and pore volume of MBG powders were determined using the Brunauer–Emmett–Teller (BET) technique at 196 °C from nitrogen adsorption and desorption isotherms.

#### 7. Transmission electron microscopic analysis

Before testing, the MBG powders were degassed at 250 °C for 3 h. Transmission electron microscopic (TEM) examination for MBG and exfoliated MoS_2_ was conducted with JEOL JEM-2010 with an operating voltage of 200 kV. The MBG powder and exfoliated MoS_2_ were dispersed in distilled water, and the solution was coated on copper grids and dried at 37 °C before the examination. During analysis, the images were captured using a charge-coupled device (CCD) camera, and selective area diffraction analyses were performed using the same system. The elemental analysis for the MBG was carried out by the energy-dispersive x-ray (EDX) analyzer.

#### 8. X-ray photoelectron spectra analysis

The x-ray photoelectron spectra (XPS, VG Scienta R3000, Uppsala, Sweden) analysis was used to confirm the surface chemical elements present in the MBG.

#### 9. UV–visible analysis

The UV–visible absorption spectroscopy of the exfoliated MoS_2_ was conducted in the range between 300 and 900 nm using Synergy HTX multimode microplate reader (BioTek Instruments Inc., USA).

#### 10. Water contact angle measurement

The water contact angle of MBG, PLGA, and PLGA–MBG–MoS_2_–Sim composites was determined with KYOWA interFAce measurement and analysis system. One sample with ten runs in different places was taken for analysis, and the average mean value was determined.

#### 11. Mechanical strength analysis

The compressive strength of PLGA–MBG–Sim and PLGA–MBG–MoS_2_–Sim composites was measured using a universal testing machine with a 10 kN load cell (Make: ZWICK, Germany and Model: ROELL Z020 20KN). The composite scaffold with a circular shape was used for the analysis. The load was applied at a constant loading rate of 0.5 mm/min until the strain reached 85%. Three samples for each scaffold composite were used to calculate the average mean mechanical strength.

#### 12. Field emission scanning electron microscopy analysis

The field emission scanning electron microscope (Make: JEOL, Japan and Model: 7610FPLUS) was used to examine the surface morphology of plain and SBF-treated PLGA–MBG–MoS_2_–Sim composites. The samples had been gold sputtered before being examined. The composite pore size was calculated using the Image J software, and the pore size data were obtained using an average mean value.

### F. Biodegradation study

The biodegradability of PLGA–MBG–MoS_2_–Sim composites was investigated by hydrolytic degradation. 1 g of PLGA–MBG–MoS_2_–Sim composite scaffold was cut in 250 mg of scaffold and used for the degradation study. 250 mg of PLGA–MBG–MoS_2_–Sim composite scaffold was soaked in 50 ml falcon tubes containing SBF and incubated at 37 °C for 28 days.^46^ Following incubation, the samples were taken out and washed with distilled water. Then, samples were dried out using the freeze-drying method and weighed again. The percentage of the degradation (WL) was calculated by the following formula:

$$W_{L}=(W_{O}-W_{1})/W_{O}\times 100,$$

where W_O_ and W_1_ represent the weight of PLGA–MBG–MoS_2_–Sim composites scaffold before and after immersion in SBF, respectively. Triplicates of PLGA–MBG–MoS_2_–Sim composites scaffold were used to calculate the mean ± standard deviation.^25^

### G. Protein adsorption study

250 mg of PLGA–MBG–MoS_2_–Sim composites scaffold were placed in a 12-well plate containing 100% ethanol. Following, the ethanol was removed from the samples, and 500 *μ*l 1× PBS (phosphate-buffered saline) was added. After 30 min, the 1× PBS were removed and 500 *μ*l of DMEM containing 10% FBS was added. Then, the samples were incubated for 3, 12, and 24 h to measure the protein adsorption efficiency at predetermined time scales.

During each incubation, the PLGA–MBG–MoS_2_–Sim composite scaffold was blot-dried and rinsed three times with 1× PBS to remove any poorly adsorbed protein on the sample's surface. Finally, the protein-adsorbed PLGA–MBG–MoS_2_–Sim composites scaffold was agitated in a radioimmunoprecipitation (RIPA) buffer for 2 h at 37 °C. Using the bovine serum albumin (BSA) protein assay technique, the Bradford standard calibration method was used to measure protein adsorption. The protein absorption was measured using a UV spectrophotometer (Shimadzu, Kyoto, Japan) at a wavelength of 595 nm.^47^

### H. Water uptake and retention ability

The dry composites of PLGA–MBG–MoS_2_–Sim were weighted (W _dry_) and immersed in distilled water for one day in 2 ml microtubes. After one day, the fabricated composite scaffolds were removed from the microtubes and placed in a Petri plate. The fabricated composite scaffolds were weighed (W_wet_) after 5 min, and the water uptake capacity was determined using the following formula:

$$\text{Water}\;\text{uptake}=(W_{\text{wet}}-W_{\text{dry}})/W_{\text{dry}}\times 100.$$

The water retention capacity of fabricated composites scaffolds was determined by transferring wet fabricated composites to a centrifuge tube with filter paper at the bottom. The setup was centrifuged for about 3 min at 500 rpm, and the fabricated composites were weighed immediately (W^1^_wet_). Finally, the water retention capacity of the fabricated composites is calculated by using the defined formula given as follows:^48^

$$\text{Water}\;\text{retension}=(W^{1}{}_{\text{wet}}-W_{\text{dry}})/W_{\text{dry}}\times 100.$$

### I. Simulated body fluid preparation

The simulated body fluid (SBF) has been formulated using the procedure discussed in Kokubo *et al.* The solution was prepared by dissolving the following materials in 350 ml de-ionized water: NaCl (4.017 g), NaHCO_3_ (0.1775 g), KCl (0.1125 g), K_2_HPO_4_·3H_2_O (0.1155 g), MgCl_2_·6H_2_O (0.1555 g), CaCl_2_ (0.146 g), and Na_2_SO_4_ (0.036 g). The pH was adjusted to 7.4 using trishydroxymethylaminomethane [(CH_2_OH)_3_CNH_2_] and 1 M HCl. Then, distilled water was added to increase the solution volume to 500 ml. The SBF was processed in a polypropylene beaker at around 36.5 ± 0.5 °C and stored at 4 °C.^49^

### J. Biomineralization study

The biomineralization study for the PLGA–MBG–MoS_2_–Sim composites was carried out in accordance with the methods described in a previous article.^46,49^ The 12.1 × 4 mm^2^ (1 g) PLGA–MBG–MoS_2_–Sim composite scaffolds were immersed in the 200 ml of SBF in polypropylene bottle and kept at 37 °C incubator for 28 days. Following, the composites were taken out from the SBF and repeatedly washed in ethanol and water to stop the further reaction. The biomineral formation potential of PLGA–MBG–MoS_2_–Sim composites was confirmed by the FTIR and XRD analysis. The morphology of the biomineral deposition was characterized by SEM analysis.^25^

### K. *In vitro* study of PLGA–MBG–MoS_2_–Sim composites

*In vitro* studies were performed in murine mesenchymal stem cells (C3H10T1/2), cultured in DMEM media with 100 units ml^−1^ penicillin–streptomycin, 10% FBS, in a 5% CO_2_ incubator at 37 °C. The mentioned PLGA–MBG–MoS_2_ composites for all cell culture experiments include PLGA–MBG, PLGA–MoS_2_, PLGA–MBG–MoS_2_, PLGA–MBG–Sim, PLGA–MoS_2_–Sim, and PLGA–MBG–MoS_2_–Sim. The fabricated composite scaffold was crushed into powder and used in the experiments.

#### 1. Cell viability

The biocompatibility behavior of PLGA–MBG–MoS_2_–Sim composites was examined using WST-1 assay kit. C3H10T1/2 cells were seeded at a density of 1 × 10^4^ in plates and incubated for 24 h at 37 °C. The fabricated composites were treated with the cells at different concentrations of 100, 200, 300, 400, and 500 *μ*g/ml. The treated cells were then incubated for 48 h. The medium was aspirated after incubation, and the cells were rinsed with a phosphate-buffered saline solution. After that, 10 *μ*l of WST-1 solution was added to the new DMEM medium and incubated for 4 h. Finally, the absorbance of the samples was measured in a multimode microplate reader at 440 and 690 nm. The results were expressed in cell viability percentage and compared to untreated cells.

#### 2. Live/dead cell staining

A live/dead cell assay was used to assess the viability of C3H10T1/2 cells after treatment with PLGA–MBG–MoS_2_–Sim composites. The cells were initially cultured in 96-well plates and allowed to grow for 24 h. Then, the cells were exposed to PLGA–MBG–MoS_2_–Sim composites at a concentration of 500 *μ*g/ml and incubated for 24 h. The staining solution of calcein and ethidium bromide was added to the cells and incubated for 15 min. The cells were then imaged using a K1-Fluo confocal fluorescence laser scanning microscope (Nanoscope systems).

#### 3. Alkaline phosphatase (ALP) activity

The ALP activity was measured in C3H10T1/2 cells as a biomarker for osteogenic differentiation. 1 × 10^4^ cells were cultured in 96 well plate and incubated for 24 h. After incubation, the cells were cultured with PLGA–MBG–MoS_2_–Sim composites at different concentrations (100, 300, and 500 *μ*g/ml) supplemented with osteogenic differentiation media (ODM) (10% FBS, 100 units ml^−1^ penicillin–streptomycin, 50 *μ*g/ml ascorbic acid, 10 mM β-glycerol phosphate, and 100 nM dexamethasone) for 1, 4, and 7 days.^50^ Cells treated with different concentrations (100, 300, and 500 *μ*g/ml) of beta-tricalcium phosphate were used as the positive control for comparison, while cells with ODM without treated samples were used as the blank. The differentiation media were changed every two days. Following an incubation period, the cells were given three PBS washes. The cells were subsequently homogenized using 100 *μ*l 0.2% Triton X-100 in 25 mM carbonate buffer with a pH of 10.3. Then, 50 *μ*l 2.5 mM MgCl_2_ and 15 mM p-nitrophenyl phosphate in 250 mM carbonate buffer were added and incubated at 37 °C for 30 min. Finally, the reaction was stopped by adding 1 M sodium hydroxide, and the absorbance of the samples was measured at 405 nm using a multimode plate reader.

#### 4. Alizarin red staining

The alizarin red assay was performed to evaluate the potential of PLGA–MBG–MoS_2_–Sim composites for osteogenesis mineral formation. The 1 × 10^4^ cells of C3H10T1/2 were cultured in 96 well plates for 24 h. Following, the cells were treated with 500 *μ*g/ml of PLGA–MBG–MoS_2_–Sim composites and incubated. The cells with 500 *μ*g/ml beta-tricalcium phosphate were used as the positive control, while the cells treated with ODM were considered blank. After incubation, the media were aspirated, and cells were washed with PBS. Then, 100 *μ*l of 10% formalin was added to cells for fixing and incubated for 30 min. Next, the formalin was removed, and 40 mM alizarin red S solution was added to cells and incubated at room temperature for 30 min in the dark conditions. Finally, the stained cells were imaged using a phase contrast biological microscope.^50^

#### 5. Bone regeneration evaluation *in vivo*: Surgical procedure and x-ray analysis

Three male New Zealand white rabbits (10–12 weeks old), each weighing 2.25 kg, were employed to examine the regeneration capacity of PLGA–MBG–MoS_2_–Sim composites (Φ 5 × 5 mm^2^) at three distinct periods of 4, 8, and 12 weeks. Intramuscular injections of ketamine (35 mg/kg) and xylazine (5 mg/kg), both administered in an aseptic manner, were used to anesthetize the animals. The hair follicles were removed on the femoral area of the left and right hind limbs, and antiseptic solutions were applied. The fascia was dissected, and the femoral condyle region was identified. A Φ 5 × 5 mm^2^ defect in the femur condyle area was created using a trephine bur, and the defects were cleaned with a physiological saline solution. Following that, the implant materials PLGA–MBG–MoS_2_ (control) and PLGA–MBG–MoS_2_–Sim (treated material) were placed on the left and right hind limbs of the femur condyle area, respectively. With this, 3.0 Vicryl sutures were used to seal the fascia, and the skin layer was stitched with nylon sutures. Later, a meloxicam injection was given to reduce the sensation of pain. After two weeks, both sutures were removed. Then, the x-ray analysis was carried out three specific times during the first, second, and third months. In all cases, the x-ray analysis was performed in the Little Paws Veterinary Clinic, Mangalore.

### L. Statistical analysis

Origin 2017 and GraphPad Prism 8.0 software were used to illustrate and analyze the data. The experiments were performed with three samples, and the values were determined in mean ± standard deviation.

## SUPPLEMENTARY MATERIAL

See the supplementary material for Figs. S1–S3 and Table S1.

## Data Availability

The data that support the findings of this study are available within the article.
